# Real-time measurement of phloem turgor pressure in *Hevea brasiliensis* with a modified cell pressure probe

**DOI:** 10.1186/1999-3110-55-19

**Published:** 2014-02-03

**Authors:** Feng An, David Cahill, James Rookes, Weifu Lin, Lingxue Kong

**Affiliations:** 1 Rubber Research Institute, Chinese Academy of Tropical Agricultural Sciences, Danzhou Investigation & Experiment Station of Tropical Crops of Ministry of Agriculture, Danzhou, Hainan 571737 China; 2 Institute for Frontier Materials, Deakin University, Waurn Ponds, Geelong, Vic 3216 Australia; 3 School of Life and Environmental Sciences, Deakin University, Waurn Ponds, Geelong, Vic 3216 Australia

**Keywords:** Cell pressure probe, Phloem turgor pressure, Phloem water relationship, Pressure flow theory, *Hevea brasiliensis*, Sap flow, Tapping

## Abstract

**Background:**

Although the pressure flow theory is widely accepted for the transport of photoassimilates in phloem sieve elements, it still requires strong experimental validation. One reason for that is the lack of a precise method for measuring the real-time phloem turgor pressure from the sink tissues, especially in tree trunks.

**Results:**

Taking the merits of *Hevea brasiliensis*, a novel phloem turgor pressure probe based on the state of the art cell pressure probe was developed. Our field measurements showed that the phloem turgor pressure probe can sensitively measure the real-time variation of phloem turgor pressure in *H. brasiliensis* but the calculation of phloem turgor pressure with xylem tension, xylem sap osmotic potential and phloem sap osmotic potential will under-estimate it. The measured phloem turgor pressure gradient in *H. brasiliensis* is contrary to the Münch theory. The phloem turgor pressure of *H. brasiliensis* varied from 8–12 bar as a consequence of water withdrawal from transpiration. Tapping could result in a sharp decrease of phloem turgor pressure followed by a recovery from 8–45 min after the tapping. The recovery of phloem turgor pressure after tapping and its change with xylem sap flow suggest the importance of phloem water relationship in the phloem turgor pressure regulation.

**Conclusion:**

The phloem turgor pressure probe is a reliable technique for measuring the real-time variation of phloem turgor pressures in *H. brasiliensis*. The technique could probably be extended to the accurate measurement of phloem turgor pressure in other woody plants which is essential to test the Münch theory and to investigate the phloem water relationship and turgor pressure regulation.

**Electronic supplementary material:**

The online version of this article (doi:10.1186/1999-3110-55-19) contains supplementary material, which is available to authorized users.

## Background

The phloem plays a central role in plant development such as nutrition transportation and inter-organ signal communication by mass transfer from source supply organ (mainly leaves) to sink demand organs (such as roots, fruits and other growth and storage organs) (Dinant and Lemoine 2010). According to the widely accepted Münch theory, turgor pressure gradient existing in sieve elements is the driving force of phloem translocation (Knoblauch and Oparka 2012). However, this pressure flow theory still requires strong experimental validation (Knoblauch and Oparka 2012)

Numerous calculations and measurements have been performed to determine the turgor pressures in plant phloem. Although most calculated turgor pressure gradients support the pressure flow theory (Housley and Fisher 1977; Rogers and Peel 1975; Turgeon 2010), experimental validations through direct measurement, particularly in trees, are insufficient due to the lack of a reliable real-time measurement method for the sink phloem (Knoblauch and Oparka 2012). The first direct measurement of phloem turgor pressure on actively growing trees was reported by (Buttery and Boatman 1964) using Bourdea and Schomeyer’s glass capillary manometer method (Bourdeau and Schopmeyer 1958). The daily height difference and the impacts of season, tapping, wounding and 2,4,5-T stimulation (Buttery and Boatman 1964, 1966, 1967) on the phloem turgor pressure in *H. brasiliensis* were examined. The phloem turgor pressure gradients change consistently due to tree stomata opening and leaf water deficit, but surprisingly the measured gradient is not along the mass flow direction. The essentially same methods were also used to measure the phloem turgor pressure of red oak (*Quercus rubrum*) by (Hammel 1968), squirting cucumber (*Ecballium elaterium)* by Sheikholeslam and Currier (1977), white ash (*Fraxinus americana)* by Susan et al. (1981) and Lee (1981). Applying a more advanced technology, Wright and Fisher (1980) and Gould et al. (2004, 2005) established a more reliable methodology with pressure probes glued onto severed aphid stylets. Hammel’s measurement in *Q. rubrum* (Hammel 1968) and Gould et al’s measurement in *Sonchus oleraceus* and *Hordeum vulgare* (Gould et al. 2005) supported the pressure flow hypothesis. However, the measured turgor pressure gradients in *E. elaterium* (Sheikholeslam and Currier 1977) and *F. americana* (Lee 1981) were not in good agreement. The phloem turgor pressures are not always strong enough to drive phloem flow and are not proportional to plant size (Turgeon 2010). Therefore, the experimental verification of the existence of the required pressure gradients from source to sink of the sieve tube has not been unambiguously tested (Knoblauch and Peters 2010).

To accurately measure phloem turgor pressure and validate Münch’s hypothesis, a reliable system will be required that can measure real-time variation of phloem turgor pressure simultaneously both from sources and sinks (Knoblauch and Oparka 2012). However, the best established aphid stylet technique is not suitable for measuring the turgor pressure at sink tissues, especially for the tree species with hard barks, because aphids usually do not feed on sink tissues (Knoblauch and Peters 2010). In addition, the needle manometer method cannot repeatedly measure the real-time turgor pressure according to its design. Basing on the early cell pressure probe technology, Milburn and Ranasinghe (1996) had tried to improve the repeatability and accuracy of the turgor pressure measurement in *H. brasiliensis* by using a high pressure pump manometer. Nevertheless, the latex meniscus deposited in the transparent tube made the observation of new meniscus difficult. It was also a very slow, unstable, and time consuming method, requiring two operators (Milburn and Ranasinghe 1996). Hence, it has not been used to measure the real-time variation of phloem turgor pressure. Although the state of the art cell pressure probe technique has been suggested to measure phloem turgor pressure in the sink tissues (Pickard 2008; Susan et al. 1981), to our knowledge, no such kind of work has been done to date.

Rubber trees (*H. brasiliensis*) are a good model plant for developing this technique and studying phloem turgor pressure. The rubber trees are abundant in the pressurized latex within their anastomosing laticifer system, resulting in its phloem turgor pressure is easier to be directly measured. The phloem anatomy and latex flow kinetics in rubber trees are more intensively studied than other laticiferous plants because of their commercial importance. In addition, phloem turgor pressure itself is a vital parameter for rubber tree production and water balance because it is the driven force for initial latex exudation and thereafter an indication for phloem water influx and the sucrose transportation from surrounding tissues. Nevertheless, changes of laticifer turgor pressure and hence latex flow regulation are still not well understood due to our inability to reliably and continuously measure real-time turgor pressures prior to and following the tapping at a single puncture.

In light of this, the state of the art cell pressure probe (Tomos and Leigh 1999) that utilises silicon oil and a pressure sensor linked to a data logger to monitor and record real-time phloem turgor pressure were developed in measuring the trunk phloem turgor pressure of mature *H. brasiliensis* trees*.* The reliability of the phloem turgor pressure probe technique was verified by measuring the phloem turgor pressure of *H. brasiliensis* subjected to tapping, height, and daily and seasonal transpiration change, and by comparing with the existing methods and results.

## Methods

### Plant materials

The rubber trees are cultivated at the experimental farm of the Chinese Academy of Tropical Agricultural Sciences (Danzhou, Hainan, China). The trees were 10 years old and tapped for 3 years with the tapping system of s/2 d3 (tapping every 3 days with half spiral) without Ethrel stimulation. The rubber trees were budded PR107 rubber clone trees unless otherwise mentioned.

### Methods

#### New system to measure phloem turgor pressure

**Figure 1:**
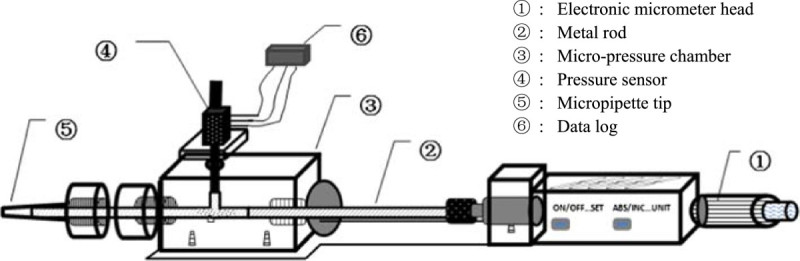
**The schematic diagram of phloem turgor pressure probe.**

The cell pressure probe was ingeniously designed to measure the turgor pressure of small and tender plant cell or the giant algae (Tomos and Leigh 1999). Due to the rubber tree bark is hard and the milky cytoplasm in laticifer cells is viscid and large in amount different to near water content of plant cell vacuoles, some modifications are required to successfully apply it in the real time turgor pressure measurement of *H. brasiliensis* phloem. Consequently the following revisions were made to construct a new phloem turgor pressure probe (PTPP, Figure 1):

1. A special highly polished micro-borer, similar to Hammel (1968), was used to facilitate the puncturing and instalment of PTPP;
2. The glass capillary was replaced with a micropipette tip having a volume of 200 μl for convenient insertion and replacement;
3. The nut at the end of the capillary was redesigned to fit the tip of a 200 μL micropipette; and
4. A thicker metal rod with a diameter of 12 mm and larger pressure chamber about 0.36 cm^3^ was used for controlling the large quantity of viscous latex influx and its interface meniscus with silicone oil.

In the turgor pressure measurement, the coarse bark firstly was slightly scraped with a blunt knife. Then, using a special micro cork borer, a small hole of approximately 2-3 mm in depth was punctured down to the hard wood of the bark to ensure the whole phloem was penetrated and measured. The cork borer was made from a hypodermic injection needle (14#) with the tip removed and modified (Hammel 1968). The borer can bring bark debris out when it removes out to lower the risk of system blockage without damaging the xylem. Following the withdrawing of the borer, the micropipette tip of the PTPP was immediately inserted tightly into the hole. The latex will flow out rapidly from many severed laticifers into the PTPP system and pressurize the silicon oil inside it. The pressure inside the PTPP was recorded with a pressure transducer (26PCFA6D, Honeywell, Germany) and a data logger (92101 Sensor-interface, Burster, Germany) to monitor the pressure change. During the measurement, the pressure inside the system can be changed while the latex exudation from the wounded laticifers was slowed, stopped or reversed by adjusting the micrometer screw at the end of the probe. The reliability of the measured turgor pressure could also be confirmed by a relaxation (suction) drop and a subsequently quick recovery of the pressure by adjusting the micrometer screw because the *H. brasiliensis* laticifer is a big reservoir of latex.

To make an accurate measurement, the system must be air tight without any leakage between the bordering of bark and PTPP. Ethylcyanoacrylate adhesive (795, Permabond Inc.) was spread at the connection of the micropipette tip and the tree bark, and then CSA-NF accelerator (Permabond quick filler setter, Permabond Inc.) was sprayed to make the adhesive cure as quickly as possible.

### Measurement of phloem turgor pressure with micropipette tip manometer

To test the reliability of PTPP, the phloem turgor pressures in some cases were also measured with the traditional micropipette tip manometer (MTM) constructed from a micropipette tip and glass capillary as was described in Milburn and Ranasinghe (1996). A small hole was bored into the bark but not into xylem with a needle having a similar diameter to the MTM tip. A manometer was then quickly inserted into the hole and sealed with little adhesive around the pipette allowing the latex exudation flowing into the MTM compressing the inside air column. Once the latex column in the capillary stabilized, which took about 4–20 min as was suggested by Buttery and Boatman (1966), the turgor pressure could be calculated from the initial and final lengths of air column within the tube according to the Boyle’s law (*P*_*1*_*V*_*1*_ *= P*_*2*_*V*_*2*_). Three to five manometers were employed but only the maximum pressure was used as the best estimation for each observation because there were sources of error falling in the observed pressure and the mean was not the best estimate of the true pressure (Buttery and Boatman 1966).

### Xylem sap flow rate

Xylem sap flow rate was measured using a FLGS-TDP sap velocity system (Dynamax, USA) according to the manufacturer’s instruction. The measurements were made at 1.3 m height from ground with a 5 cm in length probe. The data log interval was 5 min.

### Xylem tension

Xylem tension was determined with a Scholander pressure bomb (Model 3500, Soil Equipment Inc., USA). At the dusk one day before the measurement, three selected twigs at the lowermost of the canopy were sealed in a black polythene bag humidified with a wet towel inside of it overnight for full equilibrium. In the early morning of the measurement day, the twigs kept in the polythene bags were cut off and quickly transported to laboratory. Using a Scholander pressure bomb, the minimum pressures needed to make xylem sap coming out, i.e. the opposite values of xylem tension, were determined.

In the measurement, the twigs were carefully ring-barked about 2 cm at the base. The surface of the twig ending was re-cut before each reading. This could prevent the interference from latex exuding and minimize terminal blockages while pressure was applied.

### Xylem sap and latex osmotic potential

Xylem sap was collected by applying an extra pressure after the measurement of xylem tension. Latex was sampled by needle puncture method. A hole was drilled down to the bark, and then 0.5 mL of latex was collected. Latex samples were centrifuged for 30 min at 12000 rpm and the osmotic potentials of both the xylem sap and latex serum were measured with a vapour pressure osmometer (model 5520, Wescor) as Milburn and Ranasinghe (1996).

## Results

### The reliability of PTPP

**Figure 2:**
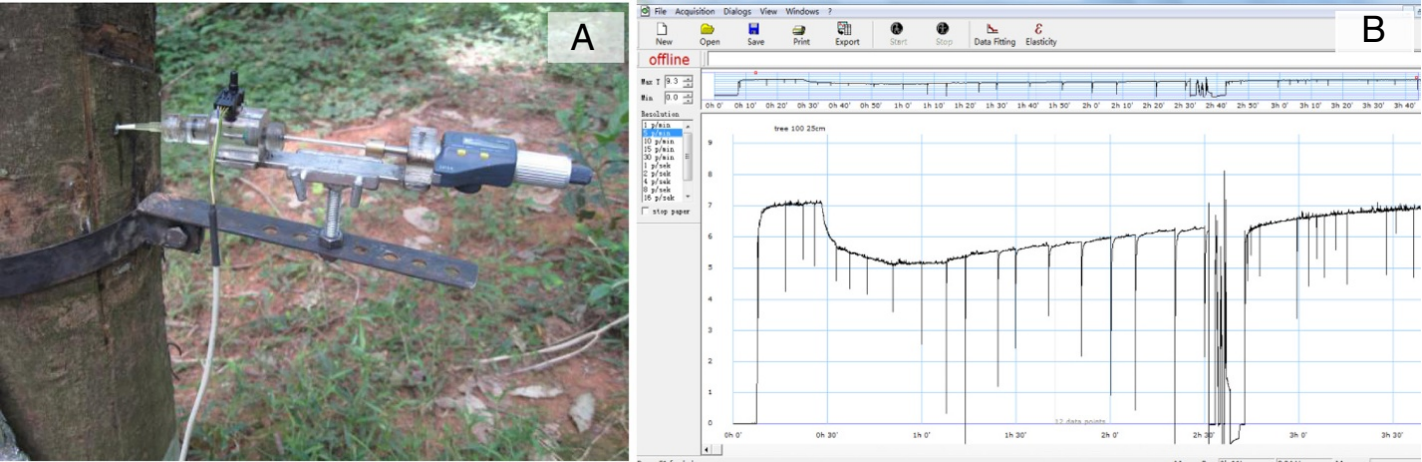
**Field setup (A) and a typical measurement (B).** The phloem turgor pressure probe was developed from the state of the art cell pressure probe with some modifications (see Methods). With a special holder, it can be easily installed and manipulated. By rotating the nut of the screw micrometer, a relaxation is made to adjust the sap/oil boundary and a distinct turgor pressure fall (spikes in the Figure 2B) could be identified. However, maintaining the meniscus at the position, the phloem turgor pressure could quickly recover to its former value. By this mean, the probe blockage and the measured pressure could be verified. If coagulation is identified, a new puncture will be required (the pressure measurement interruption in the Figure 2B).

Figure 2 showed a photo (A) and a typical result (B) for *Hevea* phloem turgor pressure measurement using PTPP. It can be seen that the PTPP is easy to be installed and manipulated. After its insertion, the measured turgor pressure will increase instantly and become balanced quickly in minutes. Then it can sensitively monitor the real time variation of phloem turgor pressure for several hours with only one puncture. When a relaxation of the pressure was performed to adjust the sap/oil meniscus by rotating the screw micrometer, a distinct turgor pressure drop could be identified. Maintaining the meniscus at the position, the phloem turgor pressure could at most times recovered quickly near to the original value, demonstrating the high sensitivity of the system. However, the system could also be blocked by latex coagulation sometimes. If the measured value drops dramatically or it cannot recover near to its original value, a new puncture and measurement is needed.

To validate the values measured with PTPP, they were compared with the values measured with micropipette tip manometer (MTM) which is a streamlined method with the most acceptable accuracy according to Milburn and Ranasinghe (1996). Table 1 showed that no significant difference could be identified between these two methods. It is therefore concluded that PTPP is a reliable technique for measuring the phloem turgor pressure of *H. brasiliensis.*

**Table 1:** **Phloem turgor pressures measured with the PTPP and MTM methods (bar)**

	Regenerated bark		Original bark	
	PTPP	MTM	PTPP	MTM
Tree1	8.67	8.71	7.91	7.86
Tree2	10.16	10.11	10.14	10.12
Tree3	6.80	6.80	7.32	7.26
Tree4	7.62	7.56	7.44	7.42
Mean	8.31^a^	8.30^a^	8.20^a^	8.17^a^

Notes: PTPP represents the phloem turgor pressure probe method and MTM represents the micropipette tip manometer method. Same superscript at upper-right of the means indicates no significant difference (*P*<0.05) could be identified between different methods. For the MTM measurements, up to five manometers were inserted to the same tree bark at the same height to the PTPP measurement, the maximum value was used as the final result. For the PTPP measurements, the phloem turgor pressure was directly read after the equilibrium pressure got.

### Indirect calculation of phloem turgor pressure in rubber trees

There is a relationship between xylem and phloem water potential balance *Ψ*_*xylem*_ = *Ψ*_*phloem*_. Therefore phloem turgor pressure could be calculated by *Ψ*_*p*(*phloem*)_ = (*Ψ*_*p*(*xylem*)_ + *Ψ*_*s*(*xylem*)_) ‒ *Ψ*_*s*(*phloem*)_ (Susan et al. 1981)*,* where *Ψ*_*p*(*xylem*)_ is the xylem tension of an overnight bagged twig, *Ψ*_*s*(*xylem*)_ is the osmotic potential of the xylem sap, and *Ψ*_*s*(*phloem*)_ is the osmotic potential of the phloem latex.

In order to further validate the measured phloem turgor pressure and also propose a method for phloem turgor pressure estimation, the phloem turgor pressure was indirectly calculated using the *Ψ*_*p*(xylem)_, *Ψ*_*s*(xylem)_ and *Ψ*_*s*(phloem)_ as was suggested by Susan et al. (1981). Measurement was made on CATAS7-33-97 virginal trees which were 6-year-old with a girth about 50 cm. Meanwhile, phloem turgor pressure was directly measured with both PTPP and MTM methods at 1.5 m height where *Ψ*_*s*(*phloem*)_ was determined. It is shown in Table 2 that the estimated turgor pressures are significantly lower than the pressures measured directly with both PTPP and MTM, which is contrary to Susan et al. (1981)‘s result where they showed that the estimated turgor was higher than the directly measured value. Therefore, the reliability of PTPP could not be confirmed by this calculation. However, as both the calculated and directly measured values are open to question and the directly measured values using PTPP and MTM are similar, we deem that the PTPP is applicable in the phloem turgor pressure measurement.

**Table 2:** **Comparisons between the calculated and directly measured phloem turgor pressures (bar)**

Tree NO.	Ψp (xylem)	Ψs (xylem)	Ψs (phloem)	Ψp (phloem)-estimated	Ψp (phloem)-PTPP	Ψp (phloem)-MTM
Tree1	−5.70	−1.99	−10.03	2.34	7.28	7.11
Tree2	−6.00	−1.42	−10.73	3.31	8.37	7.73
Tree3	−6.00	−1.87	−10.83	2.96	7.095	6.38
Tree4	−6.50	−1.74	−10.13	1.89	6.84	6.3
Tree5	−4.80	−1.97	−10.65	3.88	8.45	8.03
Tree6	−7.00	−1.92	−10.98	2.06	7.80	7.22
Mean	−6.00	−1.82	−10.56	2.74^bB^	7.64^aA^	7.13^aA^

Note: Different lowercase and capital letters at the superscripts represent the significant difference at *P* < 0.05 and *P* < 0.01 respectively. Ψp (phloem)-estimated is the phloem turgor pressure estimated with the equitation, Ψp (phloem)-PTPP means the phloem turgor pressure measured with PTPP method and Ψp (phloem)-MTM represents the phloem turgor pressure measured with MTM method. For each Tree, Ψs(phloem) is the mean of at least 3 latex samples collected from three punctures. Ψp(xylem) is the mean of minimum 3 bagged twigs and Ψs(xylem) is the mean of at least 3 xylem sap samples pressed out from the twigs used for Ψs(xylem) determination. Ψp(phloem)-PTPP is a single measurement after the PTPP was well balanced while Ψp(phloem)-MTM is the maximum value of up to five MTM measurements. Ψp(phloem)-estimated is calculated from the means of Ψp(xylem), Ψs(xylem) and Ψs(phloem) of the Trees.

### Direct measurement of phloem turgor pressure gradient along rubber trees

**Figure 3:**
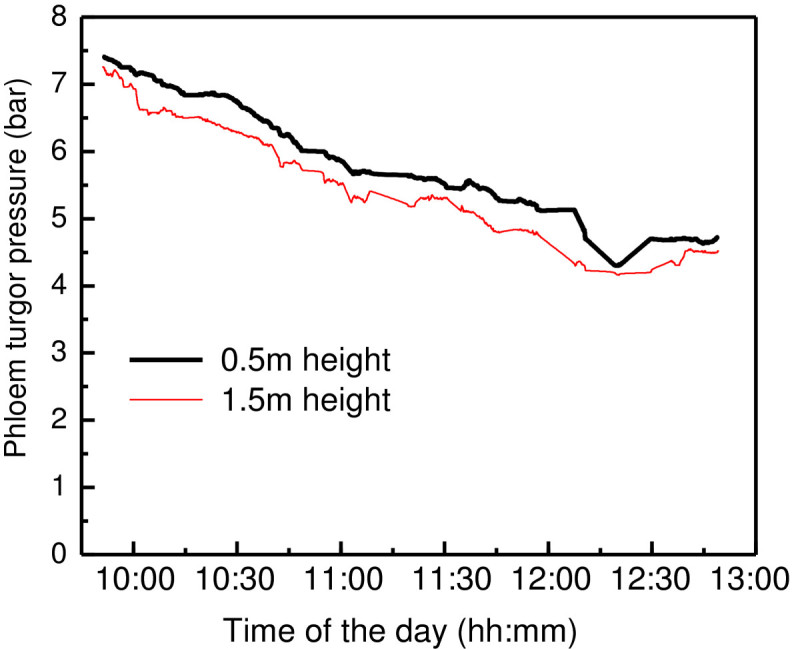
**Synchronous change of phloem turgor pressures at different heights of** ***H. brasiliensis*** **.** The measurements were made from 3 individual CATAS8-79 rubber trees, data illustrated are the simultaneously measured value at 0.5 m and 1.5 m height barks from one tree. The figure is redrawn based on the on-line measurements (see the corresponding Additional file) by deleting the relaxation validation data and some distinctly unacceptable values. Same case applies to the following figures.

To test the mass flow theory in rubber trees, the real-time phloem turgor pressures were simultaneously measured at the 0.5 m and 1.5 cm height of CATAS8-79 rubber tree bark. It is observed that the phloem turgor pressure at a lower height (0.5 cm from ground) is greater than that at an upper height (1.5 m from ground) (Figure 3 and Additional file 1). There exists a turgor pressure gradient of 0.1-0.2 bar/m from the basal to the distal end of the trunk and the phloem turgor pressure at different heights changes in a very similar pattern. These results agree to those reported by Buttery and Boatman (1964, 1966) but do not support the mass flow theory.

### Real-time measurement of diurnal turgor pressure variation

**Figure 4:**
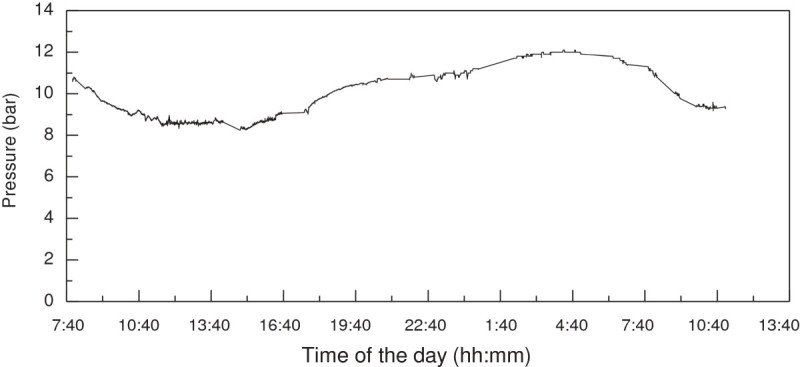
**Diurnal variation of phloem turgor pressure on PR107 rubber trees in summer.** Measurements were made in June and August, 2011 on four trees, only one typical result was shown due to they exhibited similar tendency in the diurnal rhythm.

The diurnal rhythm of phloem turgor pressure measured on a typical day (Figure 4 and Additional file 2) showed that the phloem turgor pressure was normally between 7 to 12 bar and had a daily variation in the daylight for PR107 rubber trees in summer. It reached its minimum at 14:00 to 15:00 pm and was much higher but stable from midnight to predawn. These results are in agreement with these from (Buttery and Boatman 1964; 1966) and validated the applicability of PTPP in the long time turgor pressure measurement.

**Figure 5:**
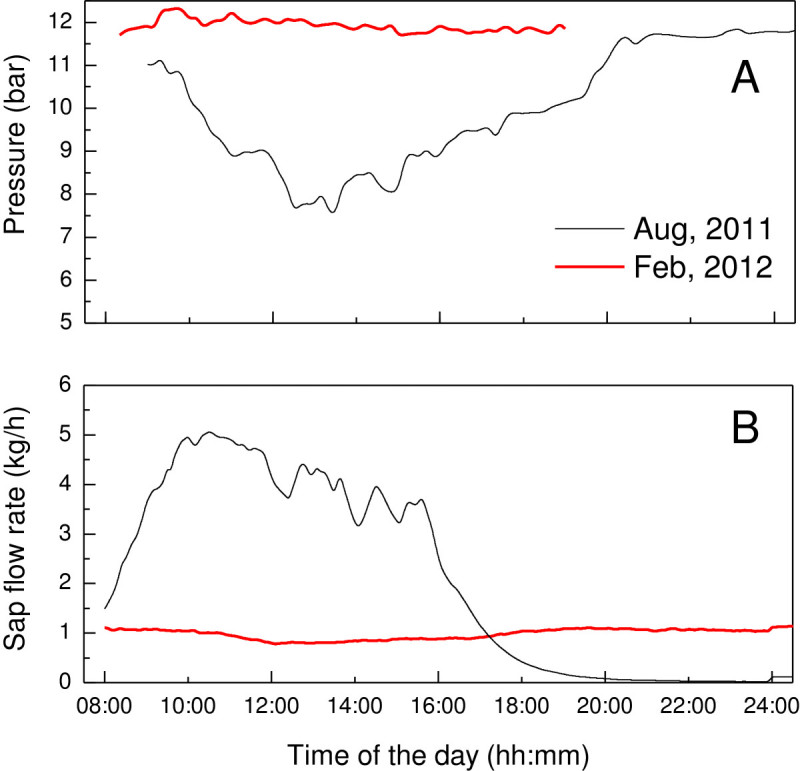
**Diurnal variation of phloem turgor pressure (A) and sap flow rate (B).** Measurements were made in both foliation (Aug, 2011) and defoliation (Feb, 2012) seasons. The phloem turgor pressure and sap flow rate are simultaneously measured on four same trees. Sap flow rates were measured at 1.3 m height with a data log interval of 5 min while real-time phloem turgor pressures were determined at about 0.6 m height. Values shown are the means from four trees.

The diurnal change phloem turgor pressure could be ascribed to the daily alteration of leaf transpiration. Although it has been suggested that the withdrawal of water from phloem to xylem by transpiration probably results in the daily variation of phloem turgor pressure (Buttery and Boatman 1964; 1966; Milburn and Ranasinghe 1996), the kinetics of phloem turgor pressure and transpiration rate have not been measured simultaneously. The reverse change of phloem turgor pressure (Figure 5A) and xylem sap flow rate (Figure 5B) on the same trees both in the foliation and defoliation seasons confirmed that. In the daylight of foliation season, the phloem turgor pressure changed reversely with the daily rhythm of sap flow rate although they were not in a synchronous fluctuation. Nevertheless, at night and in the defoliation season, where the sap flow was not very active, the phloem turgor pressure was quite high and stable.

### Real-time measurement of phloem turgor pressure after tapping

**Figure 6:**
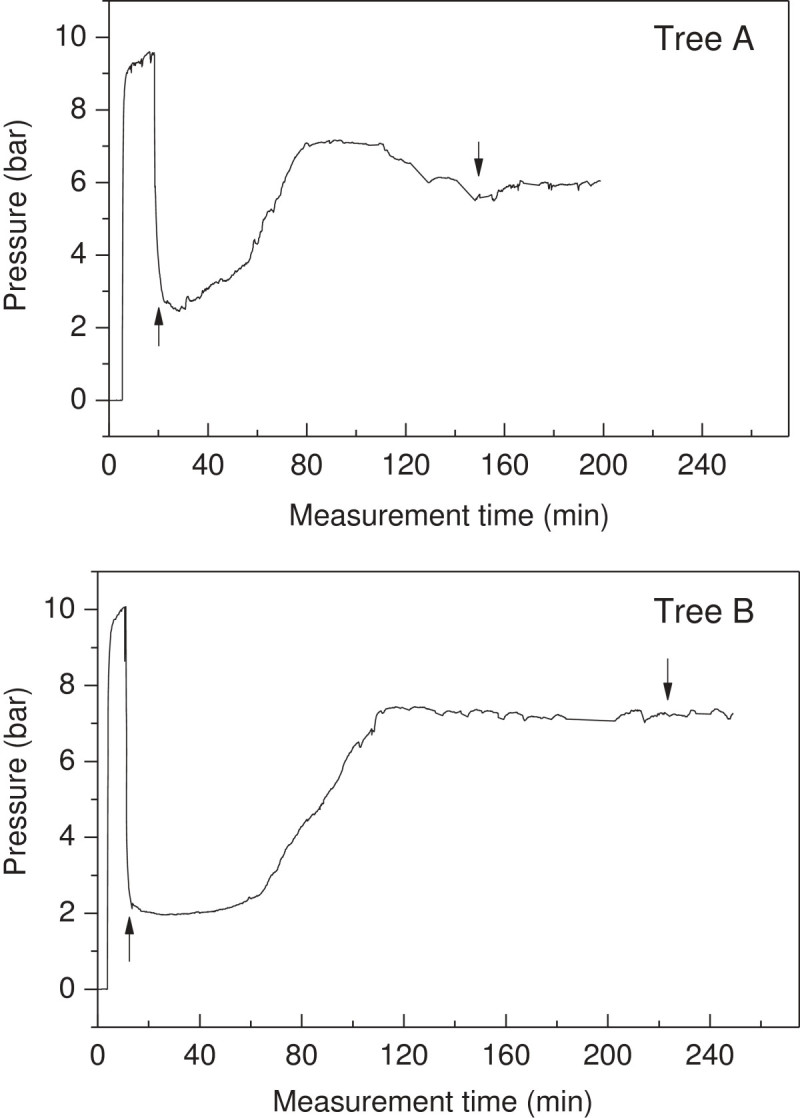
**Two typical real-time measurements of phloem turgor pressures subjected to tapping.** Tree **A** is a normal tapping tree, whilst tree **B** was subjected to 2.5% ethrel stimulation at 54 hours before the tapping. The upward arrows indicate the time of tapping and the downward arrows indicate the stoppage of latex flow.

Two typical real-time measurements of the phloem turgor pressure subjected to tapping are showed in Figure 6, Additional files 3 and 4. Once rubber trees were tapped, the phloem turgor pressure declined immediately from approximately 10 bar to 2 bar due to the opening of the served laticifer vessels. After 8–45 min, the phloem turgor pressure increased gradually as the plugging of laticifer vessels and the influx of water compensated the exudation of latex. These results further validated the accuracy of our PTPP and indicate that the plugging of laticifer vessels takes into effect 8–45 min after the tapping. The commencement time of phloem turgor pressure recovery varies with rubber tree clone, environmental conditions, and stimulation. Further experiments were set to investigate these relationships.

### Real-time measurement of phloem turgor pressure gradient after tapping

**Figure 7:**
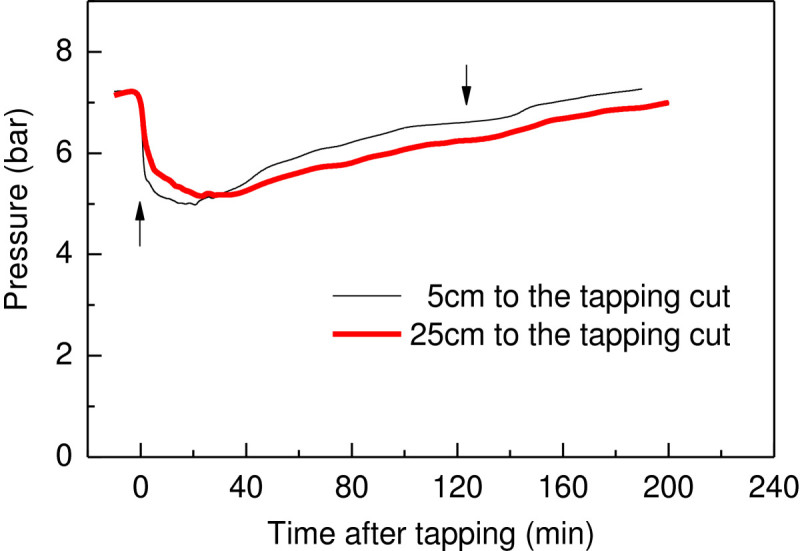
**Phloem turgor pressure variation at 5 cm and 25 cm beneath the tapping cut during latex flow.** Measurements were made on three trees, only one typical result was illustrated. The upward arrow indicates the time of tapping and the downward arrow indicates the stoppage of latex flow.

The latex flow after tapping is a two-phase hyperbolic reduction flow in which the first phase is driven by phloem turgor pressure and the second phase is driven by the latex dilution reaction (D’Auzac et al. 1989). To further validate the PTPP technique and better understand the kinetics of latex flow, the real-time variation of phloem turgor pressure was simultaneously determined at 5 cm and 25 cm below the tapping cut with two PTPPs. Figure 7 and Additional file 5 showed that the turgor pressures at the two positions change similarly with the time after tapping. The phloem turgor pressure at 5 cm beneath the tapping cut position is lower than that at 25 cm beneath the tapping cut at approximately the first 30 min after tapping. However, it becomes higher than that at the 25 cm position after that indicating the importance of osmotic dilution and laticifer plugging in the latex flow and turgor pressure recovery 30 min after tapping. This result is consistent with dendrometric measurement of the rubber tree trunk diameter (Gooding 1952; Luštinec et al. 1969) and bark contraction (Southorn 1967) at different distance below the tapping cut. It confirmed the newly developed PTPP could sensitively measure the quick change of the *H. brasiliensis* phloem turgor pressure which is an indication of the latex flow and phloem water relationship after tapping.

## Discussion

### The reliability of PTPP and its recommended application

Phloem turgor pressure is not only the initial driving force of latex flow, but also an indicator for afterward osmotic adjustment, laticifer plugging and drainage area extension after tapping (D’Auzac et al. 1989). By accurately measuring real-time variation of phloem turgor pressure, the latex flow course, laticifer plugging and overall water relationship of the laticifer system can be better understood. However, the existing methods for measuring phloem turgor pressure all could not be successfully used for the real-time measurement. The air bubble manometer (Buttery and Boatman 1966), micropipette turgor pressure manometer (Milburn and Ranasinghe 1996), and hematocrit tube manometer (Yeang 2005) are all based on the Boyle’s law. Although they are easy to operate, their accuracy is not very high and the pressure could not measure repeatedly on a single puncture.

1. The systems are filled with air which is high in compressibility and might be dissolved under pressure. They are not suitable for a high pressure measurement;
2. During the installation, bark debris and latex might block the manometer and sometime over-estimate the authentic turgor pressure;
3. Rubber latex coagulation could probably happen, leading to an under-estimation of the value;
4. As the measured values are not repeatable at a single puncture, it is sometime incorrect;
5. The manometer method is not suitable for a long time measurement. As the manometers are essentially disposable, numerous punctures will be required to measure a phloem turgor pressure dynamic. Notable disturbances to the phloem system and damage to the tree bark will be induced after few punctures;
6. It seems impossible to measure the real turgor pressure near to the tapping cut with manometers while latex flow due to the turgor pressure equilibrium could not be got in latex flow (Yeang 2005).

To make the turgor pressure measurement more accurate and repeatable, Milburn and Ranasinghe (1996) have tried to use a high pressure pump manometer based on the early cell pressure probe. However, it demonstrated neither sufficient rapidity nor precision as the system is too large and still filled with air. It was not eventually used in the real-time phloem turgor pressure.

The blockage of the systems and the prompt equilibrium has been the main challenge for the online real-time measurement of phloem turgor pressure. The real-time measurement will provide more reliable information than the currently used systems that use the highest measured pressure as the best determination (Buttery and Boatman 1964; 1966; Milburn and Ranasinghe 1996; Yeang 2005). In this study, we suggested a novel phloem turgor pressure probe based on the state of the art cell pressure probe. As the system is filled with silicon oil and constructed with a transparent micropipette, it can greatly improve the air tightness and feasibility. The dimethyl silicone oil filled in the probe is an anti-coagulant insoluble with latex. It is also low in compressibility. Thus, the PTPP could respond to turgor pressure change promptly and measure the phloem turgor pressure for several hours. The micropipette could be easily replaced after a measurement. It is cone in shape, so that the air tightness could be greatly improved. More importantly, due to the exudation from the wounded laticifers could be slowed, stopped or reversed in measurements by adjusting the sap/silicon interface, the measured turgor pressure could thus repeatedly measured and verified.

Our field measurements showed that the measured turgor pressure values and tendencies are similar to that had been reported with capillary manometer methods (Buttery and Boatman 1964, 1966, 1967; Milburn and Ranasinghe 1996; Yeang 2005). It could sensitively measure the rapid alteration of phloem turgor pressure for several hours (Figures 3, 4, 5, 6 and 7; Additional files 1, 2, 3, 4 and 5), and in accordance with tapping and daily transpiration induced trunk diameter (Gooding 1952; Luštinec et al. 1969) and bark contraction (Southorn 1967). Therefore, the novel phloem turgor pressure probe is a reliable technique to accurately measure the real-time phloem turgor pressure. Nevertheless, there are still several points need to be kept in mind: (1) it is more suitable for a long period real-time measurement, especially when the phloem turgor pressure changes rapidly. (2) The pressure probe could also be blocked by the coagulation of latex inside. Therefore, the measured value should be frequently validated by relaxation verifications. (3)The measured phloem pressure derives from a group of laticifer vessels. Therefore, it is more precisely to be the phloem turgor pressure or phloem hydrostatic pressure rather than laticifer turgor pressure. (4) There might be a possible escape of latex at the junction of laticifers themselves. (5) Latex has viscoelastic properties, whether these properties will affect the relaxation and afterward measurements requires further investigation. (6) Whether this system could be used in other plants needs to be further investigated. In spite of these, the development of cell pressure probe in this study provides an additional confidence for accurately measuring the real-time phloem turgor pressure of woody plants.

### The calculation of phloem turgor pressure

Due to phloem and xylem water relationship is in equilibrium, the turgor pressure could be calculated from other measurable parameters such as plant water potential, phloem and xylem sap osmotic potential. Many attempts have been made to indirectly calculate plant phloem turgor pressure in lacking of method to accurately measure phloem turgor pressure (Fisher 1978; Housley and Fisher 1977; Rogers and Peel 1975; Susan et al. 1981; Turgeon 2010; Tyree and Hammel 1972).

Basing on the parameters suggested by Susan et al. (1981), the phloem turgor pressure of *H. brasiliensis* was calculated and compared with the directly measured value. It is found that the estimated phloem turgor pressure is significantly lower than that of both PTPP and MTM directly measured pressures (Table 2), which is contrary to Susan et al. (1981)‘s result on *F. ameicana* where they showed that the estimated turgor was higher than the directly measured value. This is likely derived from the following reasons:

1. The assumption of *Ψ*_*xylem*_=*Ψ*_*phloem*_ is incorrect at the measurement time. Although the assumption of *Ψ*_*xylem*_=*Ψ*_*phloem*_ is certain at particular time and the phloem turgor pressure changes concomitantly with xylem tension due to radial water movement (Buttery and Boatman 1966; Molz et al. 1973), water potential between xylem and phloem is not in exact equilibrium during daylight hours (Susan et al. 1981). In addition, xylem water potential is changing with transpiration demand during the day, a time lag among phloem turgor pressure, xylem water potential and phloem water potential measurement is likely (Susan et al. 1981).
2. The three parameters were not accurately determined. For technical restrictions, the xylem tension measured for the bagged leaf was not at precisely the point where the phloem turgor pressure was measured. In addition, the osmotic potential measured from the over pressurized twigs might be contaminated by phloem resulting in a decrease of the osmotic potential. It is notable that the phloem osmotic potential in *F. ameicana* is about −20 bar according to Susan et al. (1981). However, it is normally near −10 bar in rubber trees from our measurements and other measurements (D’Auzac et al. 1989; Milburn and Ranasinghe 1996).
3. The pressure measured with PTPP is not the phloem turgor pressure of functional conducting sieve elements. In the rubber tree bark, the conducting phloem is localized in the inner most of bark (D’Auzac et al. 1989), while the turgor pressures observed by phloem turgor pressure probes derive mainly from the latex vessels, it do not necessarily reflect the conditions within the translocation system (Buttery and Boatman 1964).

Therefore, in the calculation of phloem turgor pressure, the parameters and measuring time and also plant phloem anatomy should all be carefully considered. Even, Knoblauch and Peters (2010) claimed that “the osmotic potential of exuded sieve tube sap can be measured directly, but since phloem turgor pressure depends on the difference between the osmolarities of the symplast and the apoplast solution which is generally hard to determine, turgor cannot simply be calculated from sieve tube sap osmolarity”. In this context, both the calculated and directly measured pressures existing are open to question, due to very narrow sieve elements are secluded in the complex phloem and a variety of difficulties inherent in the measurements of the parameters (Susan et al. 1981; Turgeon 2010). It is consequently vital to develop an acceptable and reliable technique to indirectly calculate or directly measure the phloem turgor pressure so as to elucidate the controversial phloem transportation mechanism.

### The phloem turgor pressure gradients in rubber trees

The widely accepted Münch hypothesis for phloem mass flow has been proposed by more than 80 years (Knoblauch and Oparka 2012). However, it is still controversial because the measured turgor pressure gradients are not always available to drive phloem flow and also it is not scale to plant size (Knoblauch and Oparka 2012; Turgeon 2010). An accurate online phloem turgor pressure measurement technique is necessary to validate or refute Münch’s hypothesis. However, the existing methods, including the best established aphid stylet method, could not be used in the real-time measurement of phloem turgor pressure at the sink tissues, especially for tree trunks. In this study, we successfully developed the state of the art cell pressure probe into the real-time measurement of rubber tree phloem turgor pressure. By simultaneously measuring the phloem turgor pressure with two PTPPs at 0.5 m and 1.5 m heights of the tree (Figure 3 and Additional file 1), we found that the phloem turgor pressure at the 1.5 m height is generally lower than that at 0.5 m height. This result is in accordance with Buttery and Boatman (1964, 1966) but contradict to the mass flow theory. Buttery and Boatman (1964, 1966) ascribed the pressure gradient contradiction with mass flow hypothesis to the anatomy of rubber tree bark. They believe that the manometer measured phloem turgor pressure mainly derives from the laticifer vessels instead of conducting sieve elements, it does not necessarily reflect the conditions of transloctary phloem system. However, due to the measured phloem turgor pressure changes with xylem sap flow rate (Figure 5) and leaf water deficit (Buttery and Boatman 1964, 1966), and girding could induce a ultimately increase of turgor pressure above the ring (Buttery and Boatman 1967), the contribution of sieve elements and laticifers to the measured phloem turgor pressure and also their equilibrium and regulation in the phloem require further studies. Although the measured turgor pressure in *H. brasiliensis* does not situate in the translocatory phloem, there is a certain relationship among them (Pickard 2008). The novel devised PTPP is useful for investigating the overall water relationship of rubber trees, particularly their phloem water exchange. Additionally, it supplies some confidence to accurately measure the phloem turgor pressure of woody plants which is essential to test the Münch theory.

### The relationship of phloem turgor pressure and phloem water balance

Phloem turgor pressure regulation has not been extensively studied. The negative relationship of phloem turgor pressure with leaf water deficit of *H. brasiliensis* (Buttery and Boatman 1964; 1966), and *F. americana* (Susan et al. 1981) suggests the water transport to the phloem laticifer vessels is directly responsible for phloem turgor pressure. Similarly, in the tapping induced latex flow course, the recovery of phloem turgor pressure could be also partially ascribed to the influx of water from surrounding tissues. The phloem turgor pressure recorded reflects the balance between fluid loss and fluid gain. In the intact laticifer fluid cytoplasm, the latex, is well equilibrated and under a turgor pressure as high as 10 bar. Upon tapping, the phloem laticifer vessels connected by anastomosis are breached and the turgor pressure of the laticiferous system near the cut will reduce to atmospheric pressure. Immediately, a turgor pressure difference of about 6.9 to 14 bar between the nearby laticiferous vessels and the cut itself will be established (Figures 6 and 7; Additional files 3, 4 and 5) (Buttery and Boatman 1966, 1967) resulting in the latex expulsion. Subsequently, latex coagulation and water influx from surrounding tissues, operating for a long while, will occur leading to a latex flow recession and turgor pressure recovery. When the tree is just tapped, the loss of fluid from the latex outflow is too great to be compensated by water inflow. Hence the turgor pressure drops very immediately after tapping. After a while (normally 8-45 min), the latex exudation from the tapping cut receded rapidly due to the gradually plugging of the severed laticifer vessels. When the rate of water inflow into the latex vessels exceeds the rate of latex exudation from the tapping cut, phloem turgor pressure rises. The gradually increase of phloem turgor pressure 8-45 min after tapping (Figure 6 and Additional files 3 and 4) is therefore a consequence of laticifer vessel water inflow accompanied by plugging that were well documented (D’Auzac et al. 1989; Frey-Wyssling 1932; Pakianathan et al. 1966; Priyadarshan 2011). The relative higher turgor pressure at 5 cm beneath the tapping cut than that at 25 cm below the tapping cut before the latex flow cease (Figure 7 and Additional file 5) is because that maximum latex dilution occurs very close to the tapping cut or even at the tapping cut itself (D’Auzac et al. 1989). Therefore, the real time measurement of rubber tree phloem turgor pressure with PTPP could better understand its latex flow and phloem water relationship. The PTPP could probably also extend to investigate the phloem water relationship and turgor pressure regulation of other woody plant trunk.

## Conclusions

A phloem turgor pressure probe based on the state of the art cell pressure probe was successfully developed to the real-time measurement of phloem turgor pressure of *H. brasiliensis.* Compared to the directly measured value, the calculation of phloem turgor pressure with Ψp_*(xylem)*_, Ψs_*(xylem)*_ and Ψs_*(phloem)*_ will under-estimate phloem turgor pressure for certain reasons. The measured phloem turgor pressure gradient is contrary to the Münch theory. The diurnal phloem turgor pressure of *H. brasiliensis* varied from 7–12 bar as a consequence of daylight change of xylem sap flow rate. Tapping could result in an immediately decrease of phloem turgor pressure. However it will gradually recover from 8–45 min after the tapping due to the influx of water from surrounding tissues and plugging of the severed laticifers. Using the newly developed phloem turgor pressure probe technique, the latex flow kinetic and rubber tree phloem water relationship could be better understood. It could probably be extended to the accurate measurement of phloem turgor pressures in other woody plants which is essential to test Münch theory, and to investigate phloem water relationship and turgor pressure regulation.

## Electronic supplementary material

Additional file 1:**Real time measurement data for Figure** 3. (PNG 143 KB)

Additional file 2:**Real time measurement data for Figure** 4. (PNG 396 KB)

Additional file 3:**Real time measurement data for Figure** 6** Tree A.**(PNG 108 KB)

Additional file 4:**Real time measurement data for Figure** 6** Tree B.**(PNG 130 KB)

Additional file 5:**Real time measurement data for Figure **7. (PNG 160 KB)

Below are the links to the authors’ original submitted files for images.

Authors’ original file for figure 1

Authors’ original file for figure 2

Authors’ original file for figure 3

Authors’ original file for figure 4

Authors’ original file for figure 5

Authors’ original file for figure 6

Authors’ original file for figure 7
