# Supplementary figures and images for: Real-time measurement of phloem turgor pressure in Hevea brasiliensis with a modified cell pressure probe
Source: Bot Stud. 2014 Feb 3;55:19. doi: 10.1186/1999-3110-55-19 (PMC5432816; doi:10.1186/1999-3110-55-19)

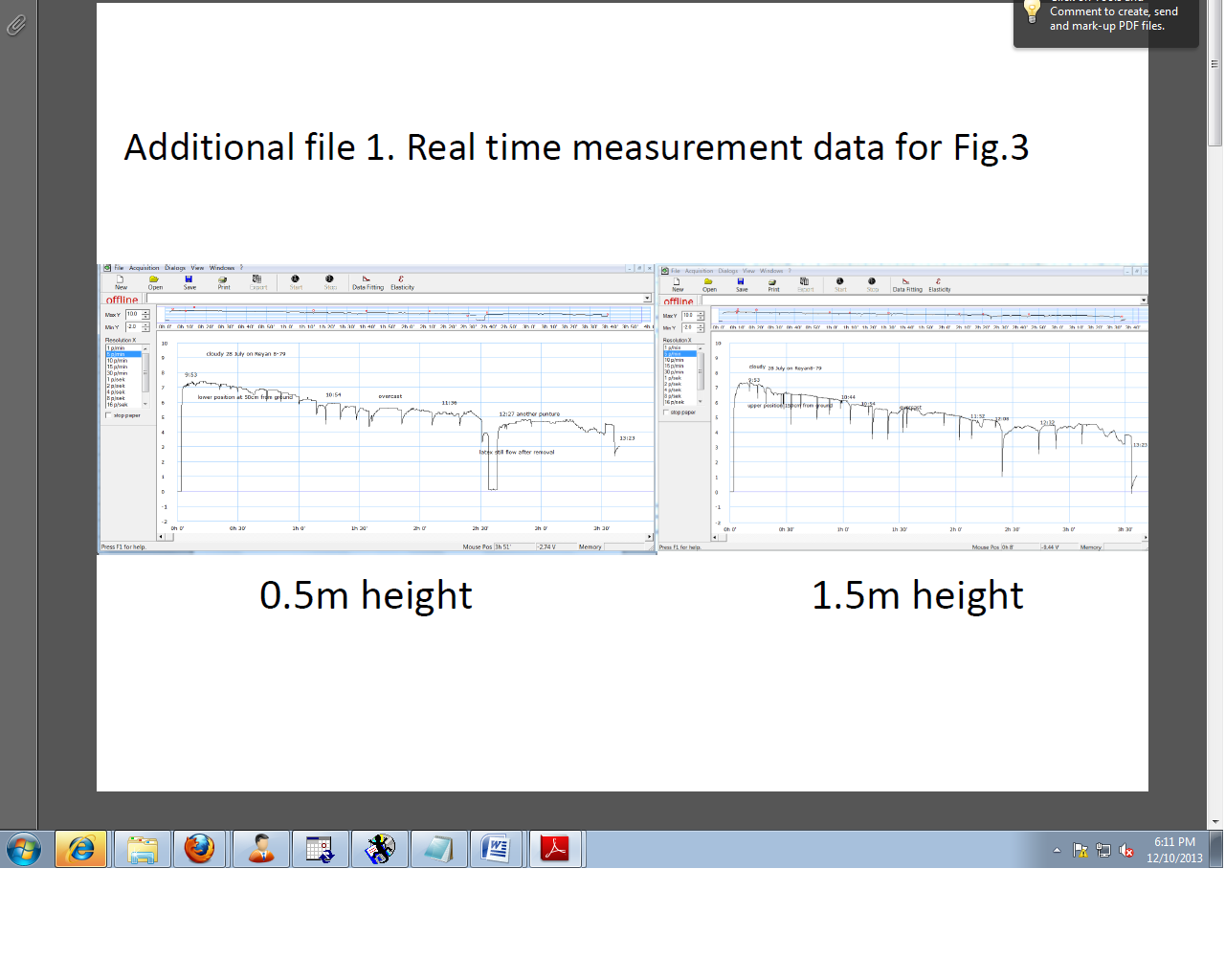

Supplement: Supplementary file 1 — Additional file 1:Real time measurement data for Figure 3. (PNG 143 KB) [file 40529_2013_62_MOESM1_ESM.png]

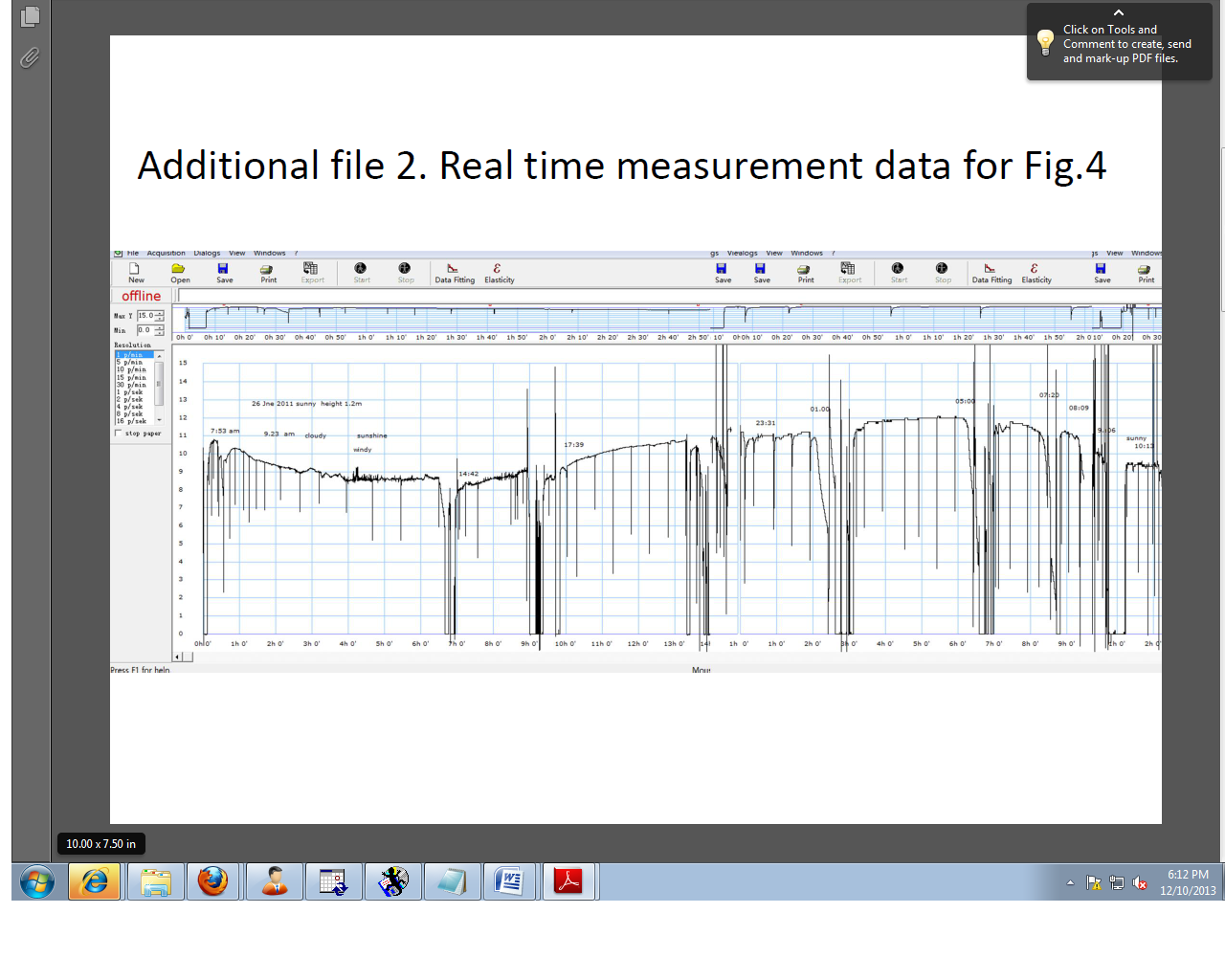

Supplement: Supplementary file 2 — Additional file 2:Real time measurement data for Figure 4. (PNG 396 KB) [file 40529_2013_62_MOESM2_ESM.png]

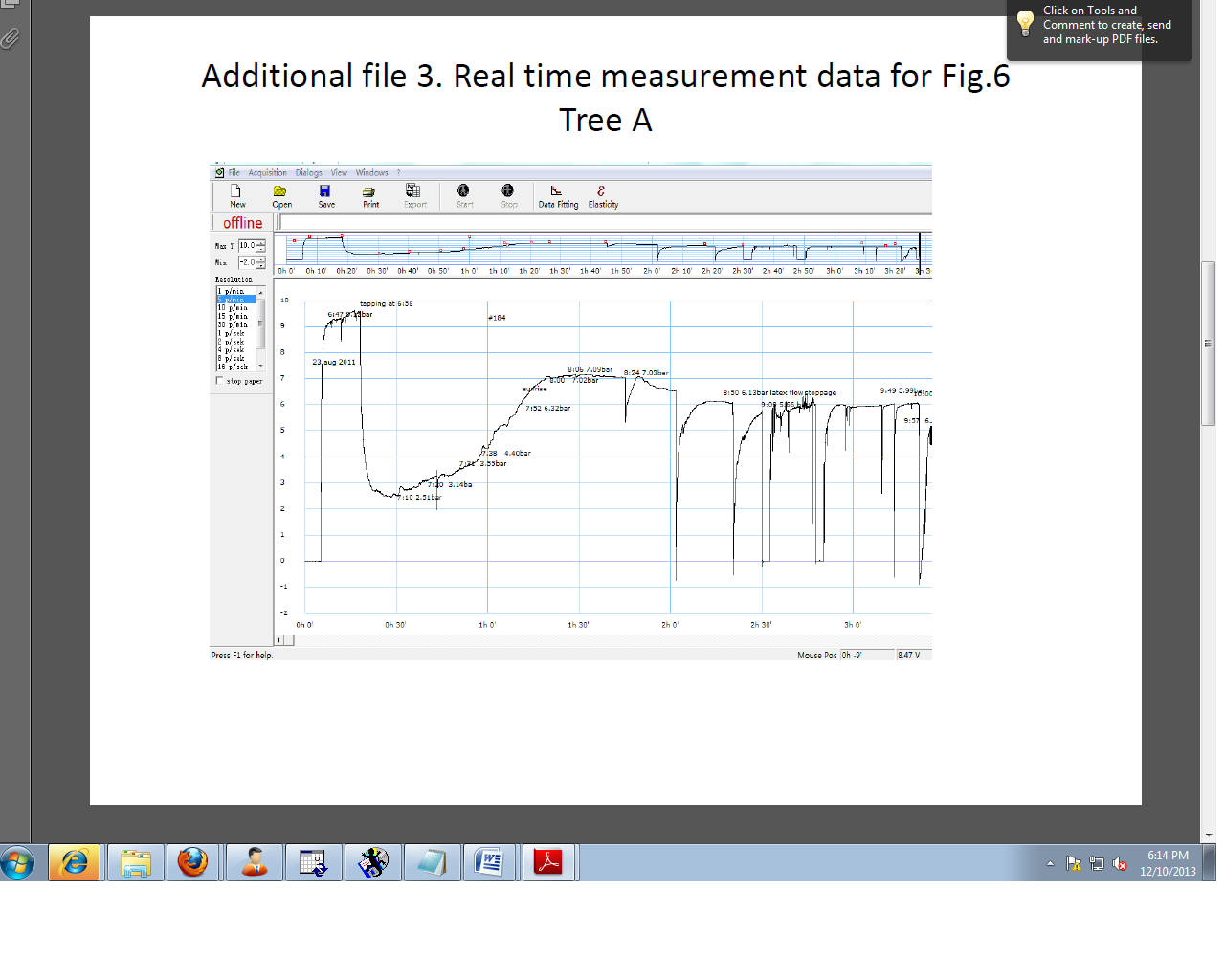

Supplement: Supplementary file 3 — Additional file 3:Real time measurement data for Figure 6 Tree A.(PNG 108 KB) [file 40529_2013_62_MOESM3_ESM.png]

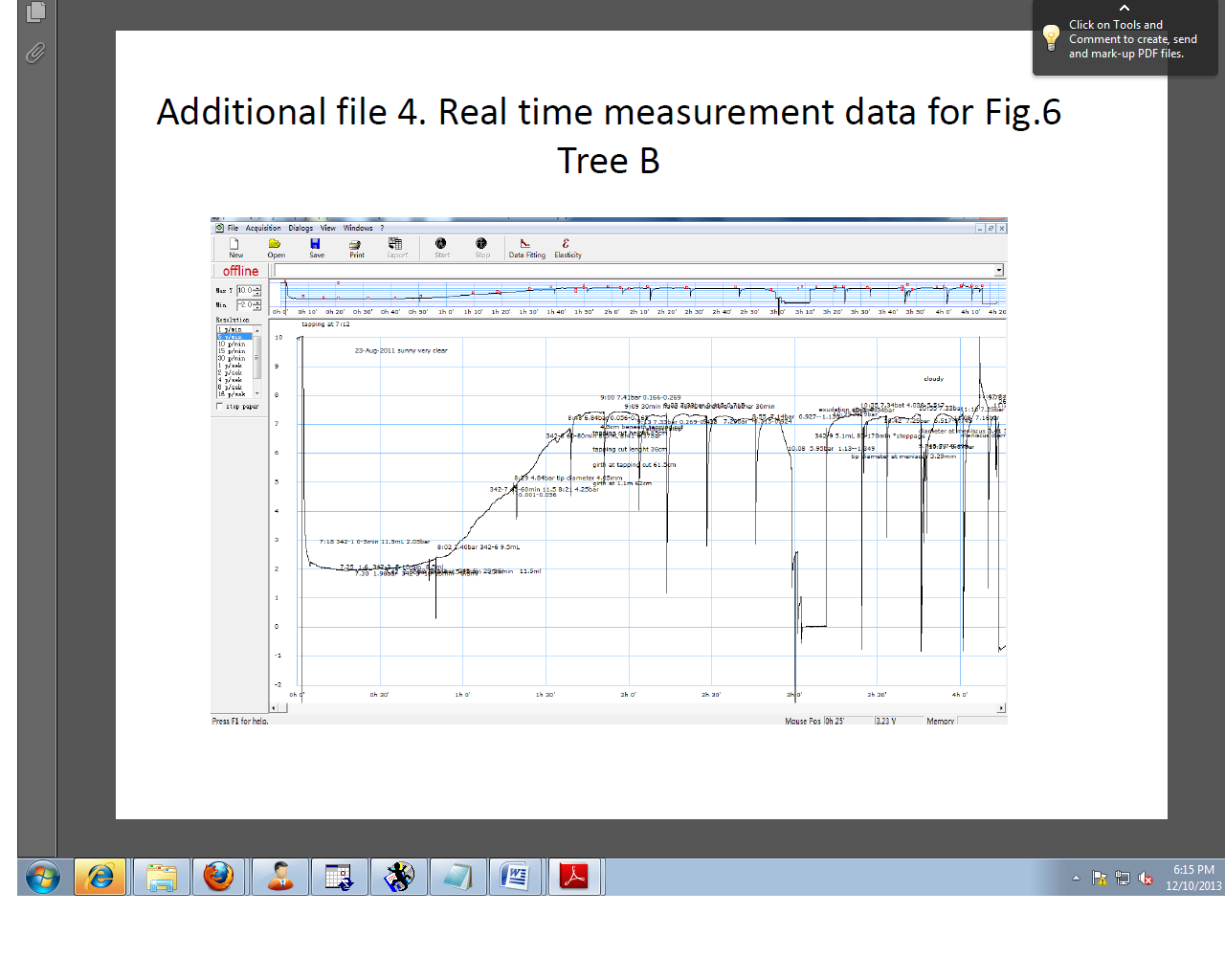

Supplement: Supplementary file 4 — Additional file 4:Real time measurement data for Figure 6 Tree B.(PNG 130 KB) [file 40529_2013_62_MOESM4_ESM.png]

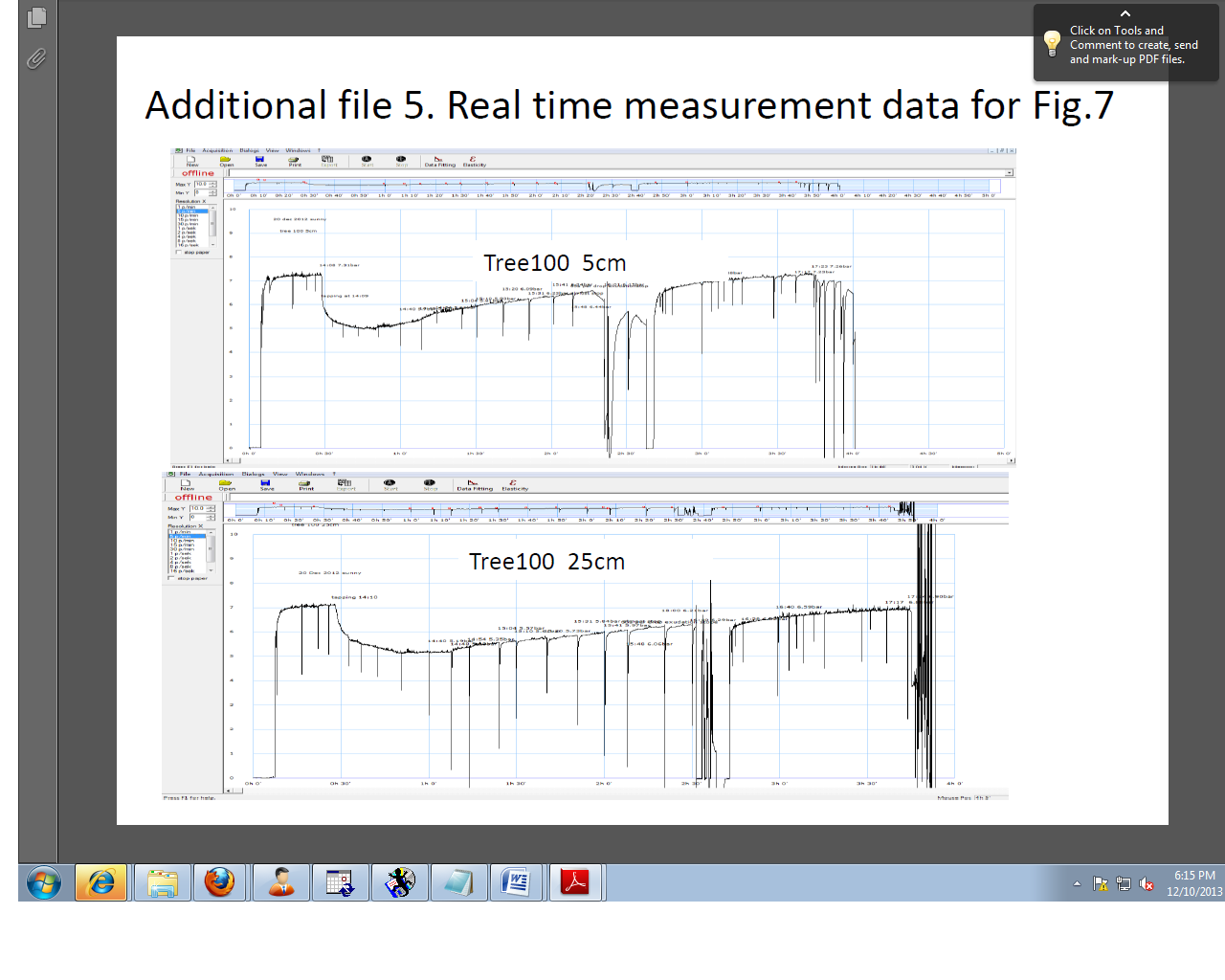

Supplement: Supplementary file 5 — Additional file 5:Real time measurement data for Figure 7. (PNG 160 KB) [file 40529_2013_62_MOESM5_ESM.png]

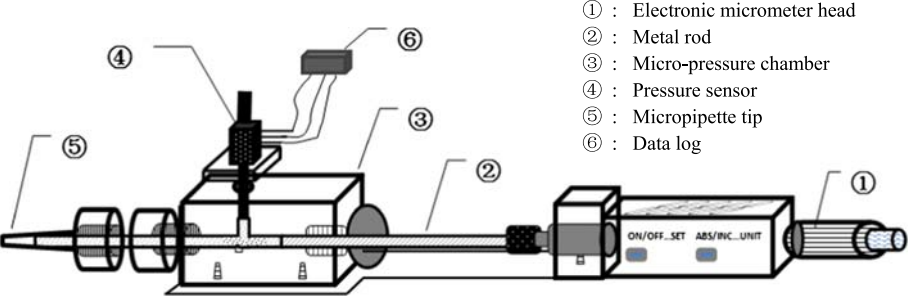

Supplement: Supplementary file 6 — Authors’ original file for figure 1 [file 40529_2013_62_MOESM6_ESM.pdf]

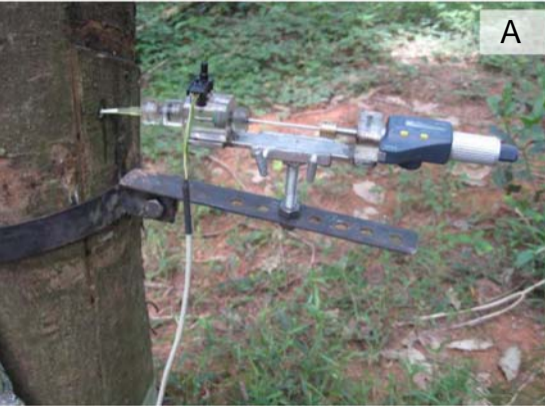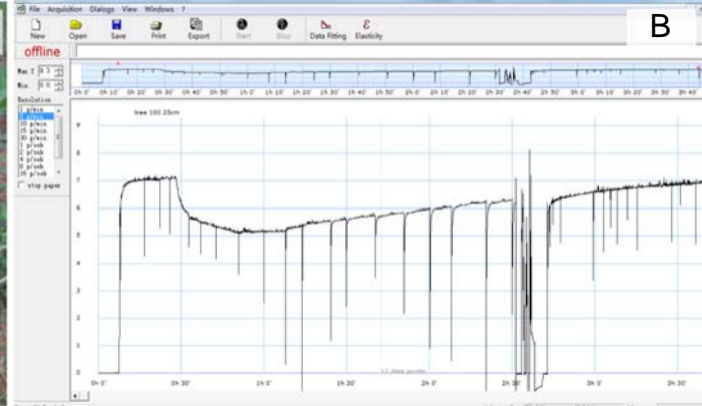

Supplement: Supplementary file 7 — Authors’ original file for figure 2 [file 40529_2013_62_MOESM7_ESM.pdf]

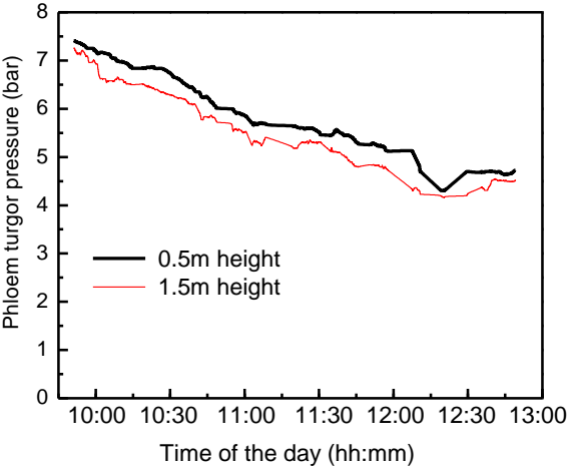

Supplement: Supplementary file 8 — Authors’ original file for figure 3 [file 40529_2013_62_MOESM8_ESM.pdf]

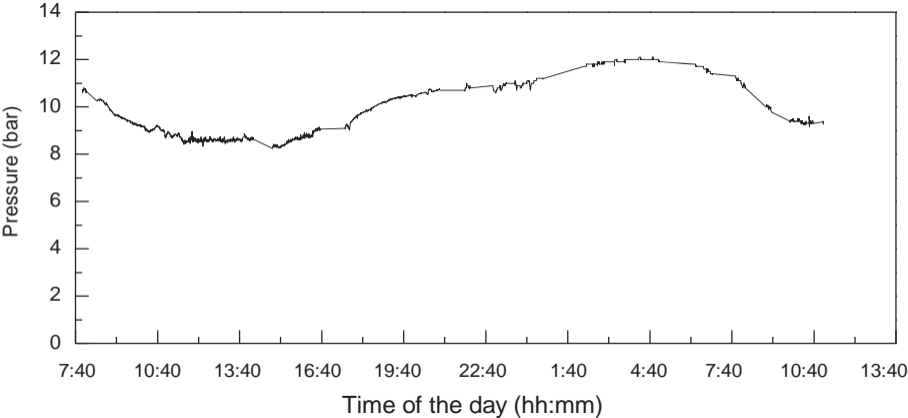

Supplement: Supplementary file 9 — Authors’ original file for figure 4 [file 40529_2013_62_MOESM9_ESM.pdf]

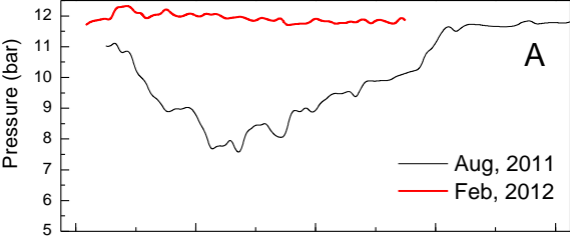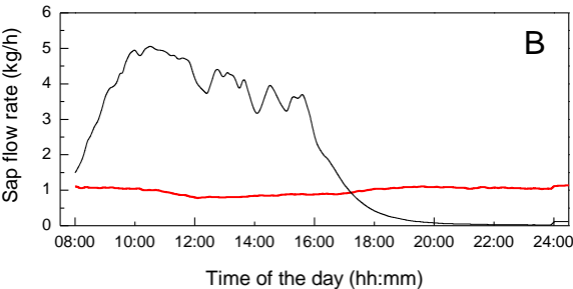

Supplement: Supplementary file 10 — Authors’ original file for figure 5 [file 40529_2013_62_MOESM10_ESM.pdf]

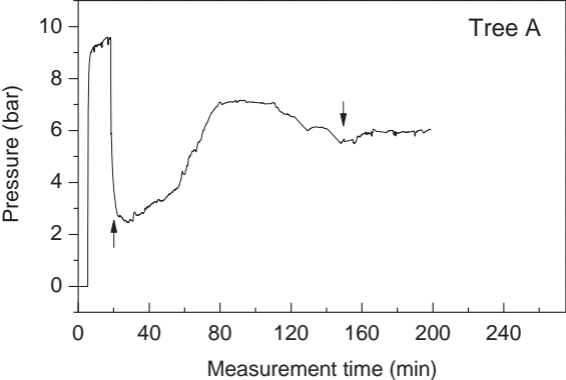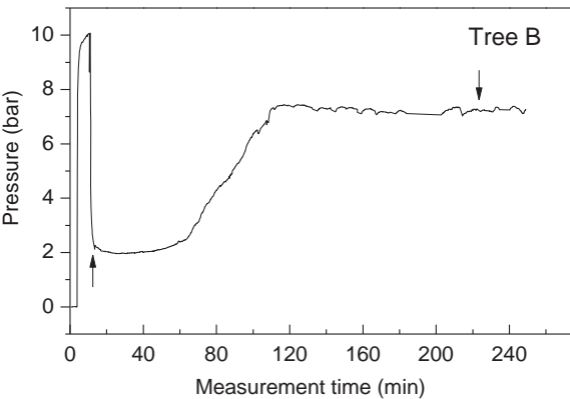

Supplement: Supplementary file 11 — Authors’ original file for figure 6 [file 40529_2013_62_MOESM11_ESM.pdf]

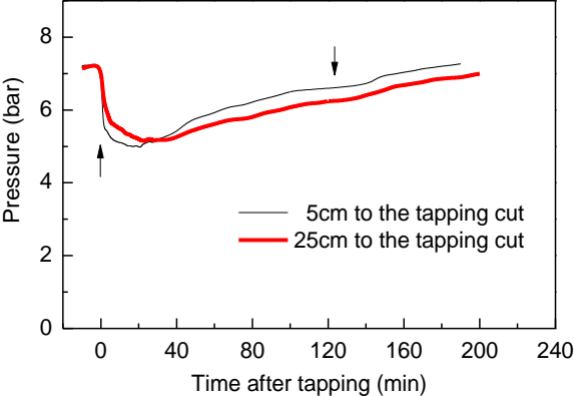

Supplement: Supplementary file 12 — Authors’ original file for figure 7 [file 40529_2013_62_MOESM12_ESM.pdf]
